# Research on Residual-Current Measurement System of Substation Considering Magnetic Shielding Effect

**DOI:** 10.3390/s24020632

**Published:** 2024-01-19

**Authors:** Jinhu Tian, Yu Xu, Yang Yang, Yingchun Zhao, Wei Man, Jingang Wang

**Affiliations:** 1 State Grid Chongqing Electric Power Company Ultra High Voltage Branch, Chongqing 400039, China; tianjh@cq.sgcc.com.cn (J.T.); xuyu@cq.sgcc.com.cn (Y.X.); zaoyc@cq.sgcc.com.cn (Y.Z.); 2 State Grid Chongqing Electric Power Company, Chongqing 400015, China; yangyang01@cq.sgcc.com.cn; 3 State Key Laboratory of Power Transmission Equipment Technology, School of Electrical Engineering, Chongqing University, Chongqing 400044, China; jingang@cqu.edu.cn

**Keywords:** residual current, magnetic shielding effect, energy saving, tunnel magnetoresistive sensor

## Abstract

Residual current is an important monitoring quantity of a power system, and a current sensor plays an important role in detecting current. The substation environment is complex. In addition to the power frequency signal, residual current also has AC and DC components. But it is also affected by the stray magnetic field of the substation. Therefore, the accuracy of the current sensor demands higher requirements. The tunnel magnetoresistive sensor has the advantages of a stable operation, high efficiency, and energy saving, but it is easily affected by the external stray magnetic field during measurements, resulting in a large error. Therefore, this paper proposes a residual-current sensing monitoring system considering the magnetic shielding effect. The root mean square error of the magnetic shielding structure is only 0.572 mA, which can effectively reduce the influence of the external magnetic field and improve the detection accuracy. At the same time, the DC measurement error is less than 1%, the AC measurement error is less than 5%, and the hybrid AC/DC error is less than 8%. It has good response ability and can accurately detect residual current.

## 1. Introduction

As a critical node for energy conversion and transmission within the power system, a substation carries the important responsibility of ensuring the safety and stability of power supply. Within substations, residual current is generated due to equipment insulation faults, grounding faults, and equipment operation faults. If this weak current cannot be accurately detected, it can directly impact the performance of the power system, personnel safety, and the reliable operation of equipment. Therefore, the issue of residual current demands high attention. Effectively detecting residual current can provide crucial information for the reliable operation of the power system [1,2,3]. The amplitude of residual current in a substation typically ranges from μA to mA, with small amplitudes. In addition to the simple power frequency AC signal, residual current also encompasses high-frequency AC components and DC components. Traditional current detection equipment, such as current transformers, can only measure AC current signals and struggle to accommodate the complex AC and DC power supply systems due to their bulky size and high costs [4,5,6,7]. The new magnetoresistive sensors, represented by TMR sensors, boast significant advantages in measuring small current signals In comparison to traditional current detection devices, they offer superior measurement accuracy and lower power consumption. However, the current use of such sensors for measurement is susceptible to interference from external stray magnetic fields [8,9,10].

The use of magnetoresistive current sensors for magnetic signal inversion makes them highly susceptible to external magnetic field interference. In an effort to enhance the measurement accuracy of these sensors, researchers have explored various designs and techniques. One such improvement is the proposal of an enhanced adaptive filtering method based on a versatile variable step size, aimed at suppressing external magnetic field interference and improving the measurement accuracy of the current sensor [11]. However, due to its reliance on array sensor arrangements, this interference suppression method is primarily suitable for high-current measurements at the ampere level, as smaller target currents may be easily overwhelmed by the useful signal. In another study [12], a U-shaped magnetic shielding was designed to enhance the measurement sensitivity of the current sensor and reduce interference from stray magnetic fields. Nevertheless, the front-end magnetic field amplification capability of this design is limited, posing challenges in accurately measuring currents at the milliampere or even microampere levels. Furthermore, Zhang et al. utilized four 3D tunnel magnetoresistive current sensors to form a sensing array, which effectively reduced measurement errors caused by external interference and improved the accuracy of the measured current values [13]. However, it is important to note that real-time magnetic field shielding was not achievable with this approach.

This paper proposes a novel multi-layer alternating magnetic ring tunnel magnetoresistive current sensor. The design incorporates alternating materials in the outer boundary shell to effectively shield external magnetic field interference. The paper begins by introducing the type and measurement principle of residual current. It then analyzes the shielding material and proposes an anti-magnetic shielding method, followed by simulation studies. Finally, the proposed sensor is experimentally verified in combination with the measurement system, leading to conclusive results.

## 2. Residual Current Measurement and Magnetic Shielding

### 2.1. Residual Current Type

The traditional residual current waveform primarily consists of a single-power-frequency 50 Hz sine-wave signal. However, with the growing presence of nonlinear equipment, such as power electronics, in substation power systems, the residual current signal has evolved to encompass various AC and DC signals, including steady DC signals [14,15,16,17]. Table 1 below illustrates some typical examples of the different residual current types.

As observed in the aforementioned table, the residual current waveform has been progressively evolving to incorporate intricate AC and DC characteristics. The current waveforms to be measured now encompass sinusoidal AC, pulsating DC, AC and DC with DC bias, steady DC, half-wave current overlaid on steady DC, and complex current waveforms. This presents new challenges for accurately detecting residual current, as it requires advanced detection methods capable of accurately capturing and analyzing these diverse waveforms.

### 2.2. Residual Current Detection Principle Based on Tunnel Magnetoresistance Effect

**Figure 1:**
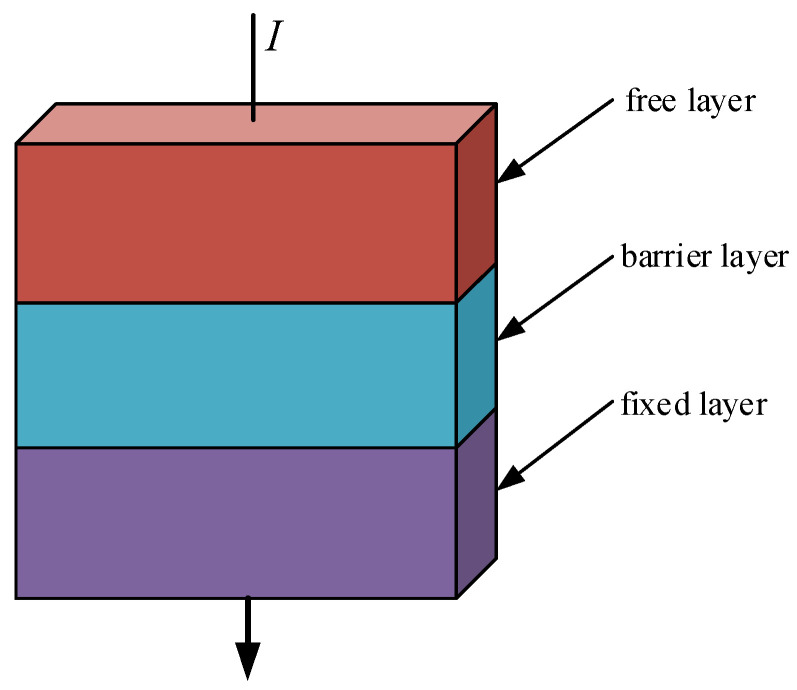
Schematic diagram of the structure of magnetic tunnel junctions.

To ensure the stable detection of complex AC and DC residual currents, the measurement system presented in this paper utilizes the tunnel magnetoresistive sensor as a crucial detection device. Its primary structure comprises a magnetic tunnel junction consisting of a ferromagnetic layer–insulator layer–ferromagnetic layer [18,19,20]. As depicted in Figure 1, the upper and lower layers’ free and fixed layers sandwich the middle barrier layer composed of insulators. The magnetic moment direction of the free layer can be altered by changes in the external magnetic field, whereas the magnetic moment direction of the fixed layer remains constant.

**Figure 2:**
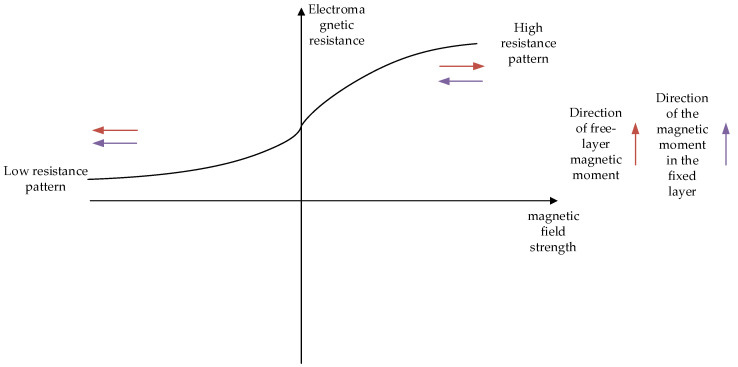
Tunnel magnetoresistive work area.

The specific principle behind residual current detection can be elucidated through the spin polarization effect. Within the magnetic tunnel structure, when the magnetization directions of the free layer and the fixed layer align in parallel, the spin electrons in the free layer can tunnel to the spin band hole state of the pinned layer through the insulating layer, resulting in the formation of electron spin polarization. This spin polarization facilitates easier current passage in the magnetic tunnel junction, thereby reducing resistance, and the structure exhibits a low-resistance state. Conversely, when the magnetization directions of the free layer and the pinned layer are anti-parallel, the spin electrons in the free layer fail to align with the spin band holes of the pinned layer in the insulating layer, leading to a reduction in current spin polarization, an increase in resistance, and the manifestation of a high-resistance state in the structure. This process is illustrated in Figure 2. During operation, the angle between the magnetic moment direction of the free layer and that of the fixed layer varies from 0° to 180°, while at the extremes of the graph corresponding to the high-resistance and low-resistance states, it reaches saturation and becomes non-operational.

The relationship between the magnitude of magnetoresistance and the angle between the magnetic moments of the free layer and the fixed layer is shown in Equation (1).

(1) $$R_{M}(\theta )=R_{P}+(R_{AP}-R_{P})\cdot \sin^{2}(\frac{\theta }{2})$$

where $R_{P}$ is the magnetoresistance when the magnetic moment directions of the free layer and the fixed layer are the same, and $R_{AP}$ is the magnetoresistance when the magnetic moment directions of the free layer and the fixed layer are opposite.

**Figure 3:**
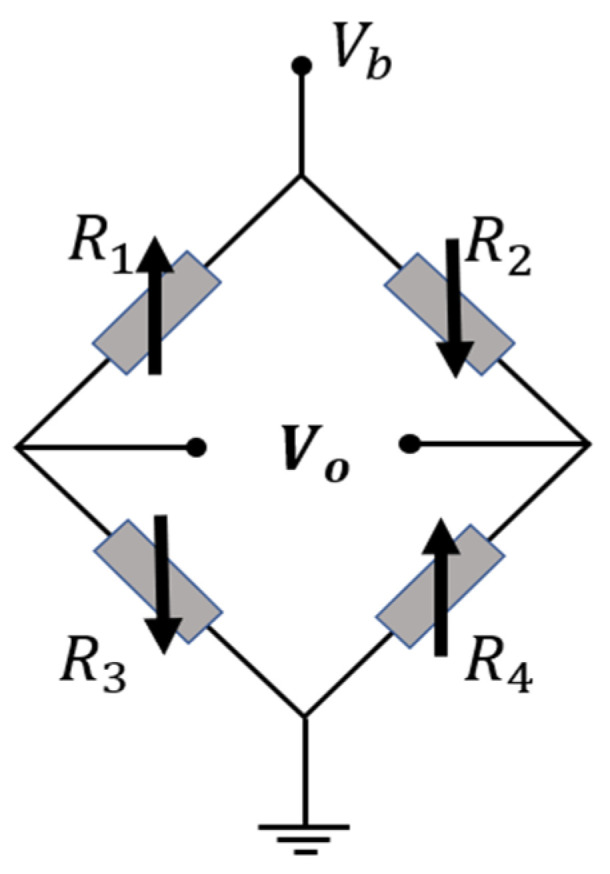
Tunnel magnetoresistive sensor’s full bridge structure.

Based on the magnetic tunnel effect, the magnetoresistance of the magnetic tunnel junction can be controlled by manipulating the magnetization direction of the free layer, enabling the conversion of magnetoelectric signals. However, in practical applications, magnetic tunnel junctions are often susceptible to hysteresis, leading to inadequate response characteristics to external magnetic fields. As a result, they fail to meet the linearity requirements, meaning the mapping relationship between the input magnetic field and the output resistance cannot be fully realized. In order to minimize the influence of stray magnetic fields from substations on the magnetic tunnel junction, the tunnel magnetoresistive sensor is typically designed as a circuit with four bridge arms. This circuit structure offers high sensitivity and is less prone to alignment errors, as depicted in Figure 3.

**Figure 4:**
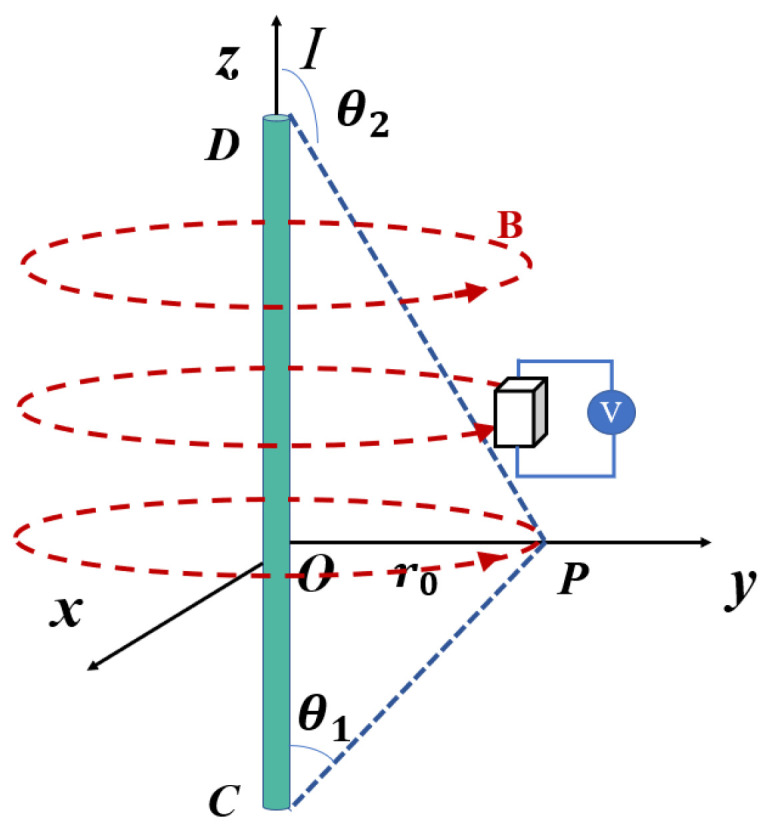
Single-wire generating magnetic filed.

After the residual current of the power equipment passes through the lead wire, according to the right-handed spiral rule, a spiral magnetic field is generated around the wire. Figure 4 shows that the current passed inside the current-carrying, long straight wire *CD* is *I*, and the distance between a point *P* in space and the wire *CD* is *r*_0_. Therefore, it can be concluded that the magnetic field generated by the wire *CD* at point *P* is:

(2) $$B=\frac{\mu_{0}I}{4\pi r_{0}}\int_{\theta_{1}}^{\theta_{2}}\sin\theta d\theta =\frac{\mu_{0}I}{4\pi r_{0}}\left(\cos\theta_{1}-\cos\theta_{2}\right)$$

When the distance between the space point *P* and the wire is much smaller than the length of the wire, the field generated at the point *P* can be simplified as follows:

(3) $$B=\frac{\mu_{0}I}{2\pi r_{0}}$$

when the length of the wire *CD* is fixed and the position of the wire and the point *P* is relatively fixed, it can be concluded that the magnetic field size *B* generated by the wire *CD* at the point *P* is proportional to the internal current value of the wire.

As noted earlier, there exists a mathematical relationship between magnetoresistance and the angle of the magnetic moment, which is influenced by the external magnetic field. When the current within the conductor fluctuates, it leads to variations in the surrounding magnetic field, consequently altering the magnetoresistance within the sensor chip. The bridge structure of the aforementioned chip can then translate these magnetic field changes into corresponding electrical signal outputs. Therefore, when the relative position of the conductor and the sensor chip remains constant, the current within the conductor can be indirectly inferred by observing the output voltage, thus achieving the objective of residual current measurement.

### 2.3. Magnetic Shielding Mechanism

In a substation, the electromagnetic environment is highly complex. It is imperative to consider not only the magnetic field generated by the sensor circuit for residual current measurement but also the stray magnetic fields emanating from the substation. These stray fields can introduce interference, causing fluctuations in the magnetoresistance of the tunnel magnetoresistive sensor, subsequently impacting the voltage output of the full bridge structure and ultimately compromising the accuracy of residual current measurements. Given the challenge of accurately obtaining geomagnetic field information, software compensation methods are unable to dynamically adapt to changes in the geomagnetic field. Hence, the utilization of hardware compensation methods becomes essential to effectively shield against magnetic field interference. Electromagnetic shielding technology proves to be an effective means of suppressing electromagnetic noise propagation within the spatial confines of the substation. The underlying principle involves severing the propagation path between the noise source and the sensor [21,22,23,24]. Electromagnetic shielding encompasses three main types: electric field shielding, magnetic field shielding, and electromagnetic field shielding. Among these, magnetic field shielding imposes the most stringent material and structural requirements. The fundamental principle of magnetic shielding lies in the partial penetration of external magnetic fields through air and ferromagnetic materials. Due to the considerably higher permeability of magnetic shielding materials compared to air, the magnetic flux predominantly passes through the ferromagnetic material. The analysis of low-frequency magnetic field shielding is typically approached through magnetic circuit theory. Suppose that the magnetic flux between two points a and b is Φ*_m_*, the magnetoresistance is R*_m_*, and the magnetic potential difference is U*_m_*, then according to the magnetic circuit theory, there is the following:

(4) $$U_{m}=R_{m}\Phi_{m}=\int_{a}^{b}Hdl$$

(5) $$\Phi_{m}=\int_{S}BdS$$

(6) $$R_{m}=\frac{\int_{a}^{b}Hdl}{\int_{S}BdS}$$

where *H* is the magnetic field intensity, *B* is the magnetic induction intensity per unit area, *S* is the normal area of the magnetic circuit, and *l* is the length of the magnetic circuit.

If the magnetic field is uniform and the magnetic circuit area is also uniform, there is the following:

(7) $$R_{m}=\frac{Hl}{BS}=\frac{l}{\mu S}$$

where *μ* is the permeability of ferromagnetic material.

According to Formula (4), when the magnetic potential difference U*_m_* between two points in the magnetic field is constant, the smaller the magnetoresistance R*_m_*, the larger the magnetic flux Φ*_m_*. To enhance the shielding effectiveness against stray magnetic fields and minimize their impact on the magnetic field generated by residual current, it is crucial to carefully select appropriate materials. Magnetic materials can be broadly categorized into hard magnetic materials and soft magnetic materials. Hard magnetic materials are challenging to magnetize, and once magnetized, they retain a certain level of magnetism even after the removal of the external magnetic field. Consequently, hard magnetic materials are typically used for permanent magnets, but they exhibit higher magnetic losses.

On the other hand, soft magnetic materials possess small hysteresis, making them easily magnetizable with minimal magnetic losses. By utilizing soft magnetic materials, the direction of magnetic flux can be altered, resulting in concentrated magnetic fields. This enhances the sensor’s sensitivity and resolution in detecting magnetic induction intensity. Moreover, soft magnetic materials can be employed as magnetic shields to effectively block external interference magnetic fields, ensuring that the detection process remains unaffected by them. According to Equation (7), R*_m_* is inversely proportional to *μ* and *S.* Therefore, in order to ensure the shielding effect, the shielding body needs to have a smaller R*_m_*. To effectively reduce the impact of external stray magnetic fields on the measurement accuracy of the tunnel magnetoresistive sensor’s residual current, it is essential to select ferromagnetic materials with high permeability. This ensures that the majority of the magnetic flux can traverse through the shielding body. Nickel–iron alloy, permalloy, silicon steel sheets, and other similar materials are well-suited for serving as the shielding body due to their favorable properties. Moreover, increasing the thickness of the shielding layer appropriately can endow the material with a higher magnetic saturation strength, thereby enabling a larger external magnetic field to pass through. In the presence of strong low-frequency magnetic fields, the use of a multi-layer shielding body can effectively safeguard the current sensor. This paper focuses on the design of a magnetic shielding shell tailored to the material properties, aiming to mitigate the influence of external stray magnetic fields on the accuracy of residual current measurement by the tunnel magnetoresistive sensor.

## 3. Design of Residual Current Detection System with Magnetic Field Shielding

### 3.1. Design of Magnetic Field Shielding Model

Based on the aforementioned analysis, it is evident that the tunnel magnetoresistive sensor demonstrates high sensitivity to magnetic fields, enabling the indirect measurement of current by detecting the magnetic field generated by the energized conductor. Consequently, when assessing the residual current of the substation, external interference magnetic fields may elevate the measurement error of the current sensor. Therefore, in order to enhance the sensor’s accuracy in measuring residual current, it becomes imperative to shield against external electromagnetic interference.

**Figure 5:**
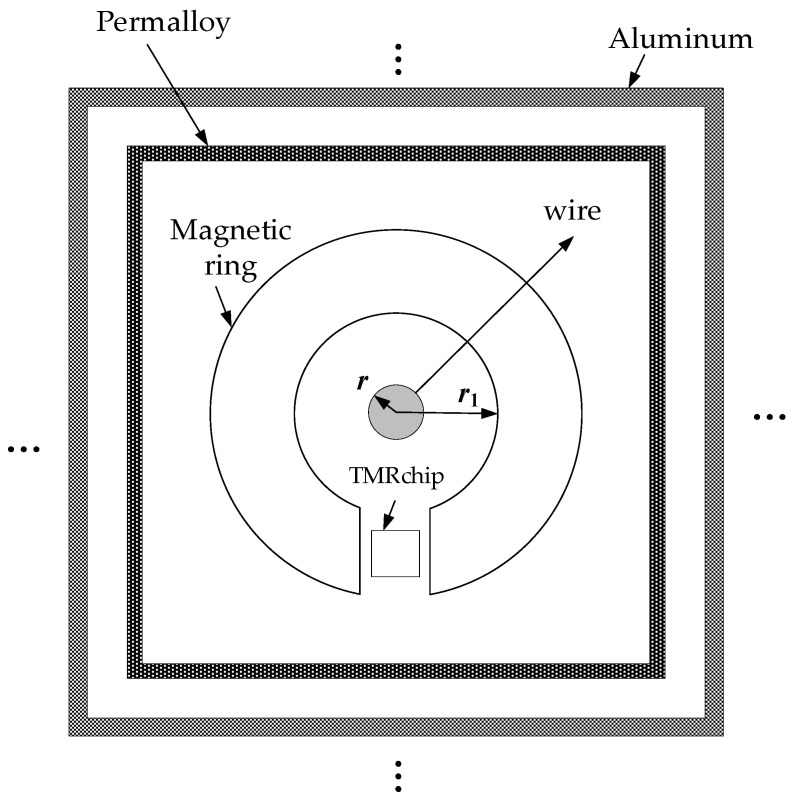
Shielding structure schematic.

Currently, research on anti-interference for magnetic interference typically employs a single-stage magnetic ring structure, with the sensor chip embedded in the air gap to concentrate the target magnetic field. This magnetic ring structure provides a certain degree of shielding against magnetic interference. Generally, materials with high permeability exhibit stronger magnetic-field-shielding performance, thereby achieving a superior shielding effect. Moreover, the effectiveness of magnetic field shielding can be enhanced through the combination of different materials. The integration of high-conductivity materials and high-permeability materials yields high efficiency in shielding. In this paper, a multi-layer magnetic ring tunnel magnetoresistive residual current sensor structure is proposed in Figure 5. This structure utilizes a multi-layer alternating metal shielding shell to counteract disruptive magnetic fields. The ellipsis indicates the potential addition of more shielding layers for practical application. The inner layer employs iron-based amorphous alloy material with high permeability, while the outer shell consists of a copper shell. Simultaneously, measures are taken to prevent the magnetic field attenuation of the current from being measured, and the magnetic ring is retained inside to reinforce and measure the magnetic field to be measured. The number of overlapping layers of the shielding layer can be specifically designed according to the situation. This innovative design empowers the sensor to ensure the accuracy and stability of the measurement while reducing interference. The configuration of a multi-layer alternating shielding shell further enhances the shielding effect of the sensor on the interference source, thereby improving the anti-interference capability of the entire system.

The front-end structure of the multi-layer alternating magnetic ring tunnel magnetoresistive residual current sensor comprises a multi-layer alternating metal shielding shell and a magnetic ring. The sensor chip is embedded in the air gap of the magnetic ring and connected to the signal processing unit. This structure effectively shields external magnetic interference through the multi-layer alternating metal shielding shell, while utilizing the magnetic ring to amplify the target magnetic field, which is then measured by the sensing chip. Subsequently, the magnetic field signal is transformed into a voltage signal via the Wheatstone bridge of the tunnel magnetoresistive sensor structure, and the sampled voltage signal is processed in the signal processing unit to derive the measured current information. The key advantage of this structure lies in its ability to effectively shield external magnetic interference without requiring specific information about the interference magnetic field. Additionally, the presence of the shielding shell ensures that the target magnetic field is not attenuated. By leveraging a closed-loop magnetic circuit, different magnetic circuit media can be recognized as a series magnetic circuit, enabling the parallel magnetic circuit analysis of adjacent media.

Based on the previous materials, it is evident that the magnetic circuit with higher permeability in the magnetic ring exhibits lower magnetoresistance, resulting in the concentration of the magnetic flux generated by the target current within the magnetic ring for amplification. The outer layer of the magnetic ring comprises a multi-layer alternating metal shielding shell, which features a combination of high-conductivity and high-permeability materials. The high-conductivity layer is capable of producing an eddy current effect, effectively attenuating the external interference magnetic field and excluding the interference magnetic field from the magnetic circuit generated by the target current. This signifies the realization of eddy current elimination for the alternating magnetic field generated by high magnetic interference. Furthermore, the low-reluctance magnetic flux path created by the high-permeability material guides the external interference magnetic induction lines to travel along the wall of the high-permeability layer, thereby achieving the diversion of an external quasi-static interference flux. The calculation of the external electromagnetic propagation coefficient is shown in Equation (8). Any combination of dielectric constant, permeability, and conductivity that increases the attenuation constant can suppress the interference of the external magnetic field. The difference in the combination series and the size of the shielding shell medium bring different media combinations. When the appropriate combination form is adopted, the shielding layer can not only suppress the leakage of the magnetic field generated by the target current, but also improve the signal-to-noise ratio of the weak-current-induced magnetic field measurement.

(8) $$\tau =\sqrt{j\omega \mu (\sigma +j\omega \varepsilon )}=\alpha +j\beta$$

In Equation (8), *β* is the phase constant. The magnetic ring material gathers the target current magnetic field, and the permalloy with high permeability is selected. The multi-layer alternating metal shielding shell is composed of a high-conductivity material and high-permeability material. Considering the weight and shielding effectiveness, the alternating material of permalloy and pure aluminum is selected.

### 3.2. Magnetic Shielding Simulation Analysis

**Figure 6:**
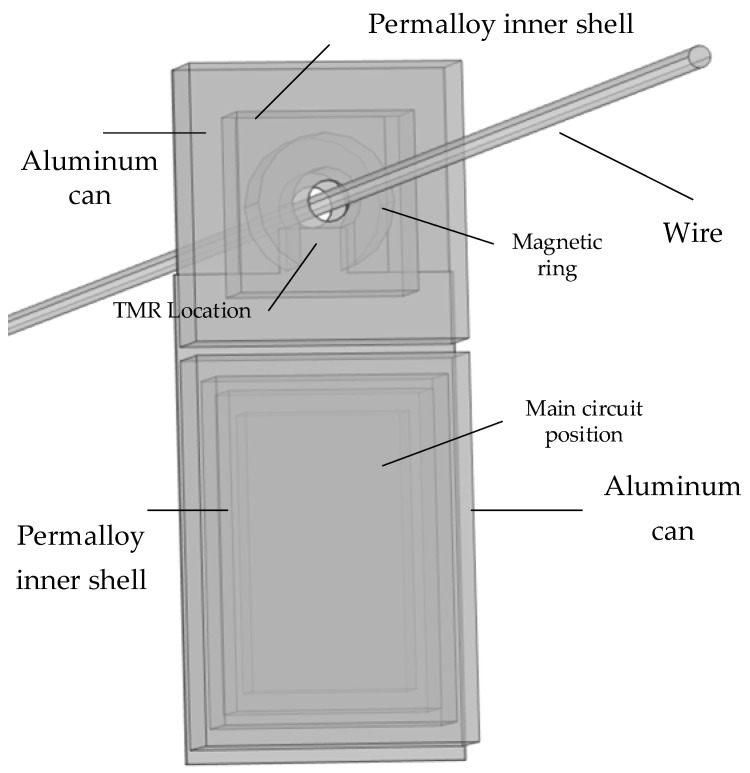
Simulation schematic.

To evaluate the measurement performance of the designated structure and determine the optimal number of overlapping layers, a finite element model of the multi-layer alternating metal shielding shell and the magnetic ring was established using COMSOL Multiphysics software. As depicted in Figure 6, the magnetic capacity and anti-magnetic interference performance of the multi-layer alternating magnetic ring tunnel magnetoresistive current sensor were thoroughly assessed and analyzed. Additionally, Figure 6 aids in identifying the suitable number of overlapping layers.

Model materials and size settings are shown in the Table 2 and Table 3.

In the tables, the material of the wire is copper, *r* is the radius of the wire, r_1_ is the inner radius of the magnetic ring, h is the space height of the magnetic ring, d is the air gap width, and th overlaps each layer thickness.

In order to intuitively evaluate the magnetic concentration ability and anti-magnetic interference performance of the multi-shield tunnel magnetoresistive magnetic ring sensor, it is necessary to establish the magnetic measurement evaluation index of the sensor and define the magnetic field magnification MA and the magnetic measurement relative error ER, respectively. Here, MA is the absolute-value ratio of the magnetic field in the direction of the sensitive axis detected by the multi-shielded magnetic ring structure and the single-wire structure at the air gap point, which is defined as follows:

(9) $$\mathrm{MA}=\frac{\left|B_{A}\right|}{\left|B_{0}\right|}$$

where *B_A_* is the sensitive direction magnetic field vector detected by the multi-shielded magnetic ring structure at the air gap point, and *B*_0_ is the magnetic field vector of the single-wire structure at the air gap point. The larger the magnification, it is proved, the more effectively it can aggregate and amplify the magnetic field. The definition of *ER* is as follows:

(10) $$ER=\frac{\left|B_{e}-B\right|}{\left|B\right|}$$

where *B* is the magnetic induction intensity detected by the air gap point without interference, and *B_e_* is the magnetic induction intensity detected by the air gap point with interference. The smaller the relative error, the smaller the difference between the measured results of the sensor and the actual magnetic field value, and the higher the measurement accuracy and anti-interference performance.

**Figure 7:**
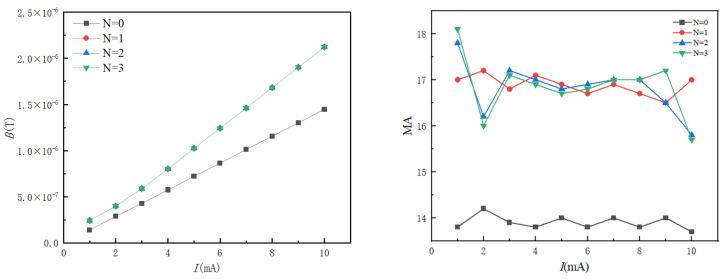
Comparison of magnetic field strength and MA variation with current for different numbers of interleaved layers.

To verify the effectiveness of the proposed structure and select the appropriate number of overlapping layers for simulation analysis, when N = 0, the sensor structure only has a magnetic ring without a metal shielding shell; when N = 1, the shielding shell consists of a layer of copper and permalloy; when N = 2, it is a two-layer copper and permalloy; when N = 3, it is a three-layer copper and permalloy. The magnification of different overlapping layers N is simulated and analyzed. In the current line to be measured, the current value starts from 1 mA and gradually increases to 10 mA with a step size of 1 mA through 50 Hz AC current I. The comparison between the magnetic field intensity at the air gap point and the magnification of different overlapping layers is shown in Figure 7.

Figure 7 illustrates a direct correlation between the increasing current value to be measured and the magnetic field intensity at the air gap point, displaying a positive linear relationship between the two variables. In comparison to the single magnetic ring structure, it is evident that the magnetic ring structure with a shielding layer yields a larger magnetic field magnification, thus enhancing the magnetic field measurement accuracy and sensitivity. The introduction of a shielding layer, formed by the multilayer alternating metal structure, establishes a complex magnetic flux path, facilitating the improved gathering and guidance of the magnetic field. Consequently, the magnetic field intensity measured at the air gap point is significantly amplified, leading to a higher magnetic field magnification. This enhancement allows for a more accurate measurement and detection of current signals of the same magnitude by the sensor. Furthermore, as the number of alternating layers increases, there is observed consistency in the magnetic field intensity at the air gap point. This suggests that within a certain range, the increment in the number of alternating layers does not yield a substantial change in the magnetic field magnification. This characteristic offers flexibility in the design and manufacturing process, enabling optimization according to specific application requirements while maintaining a certain shielding effect.

**Figure 8:**
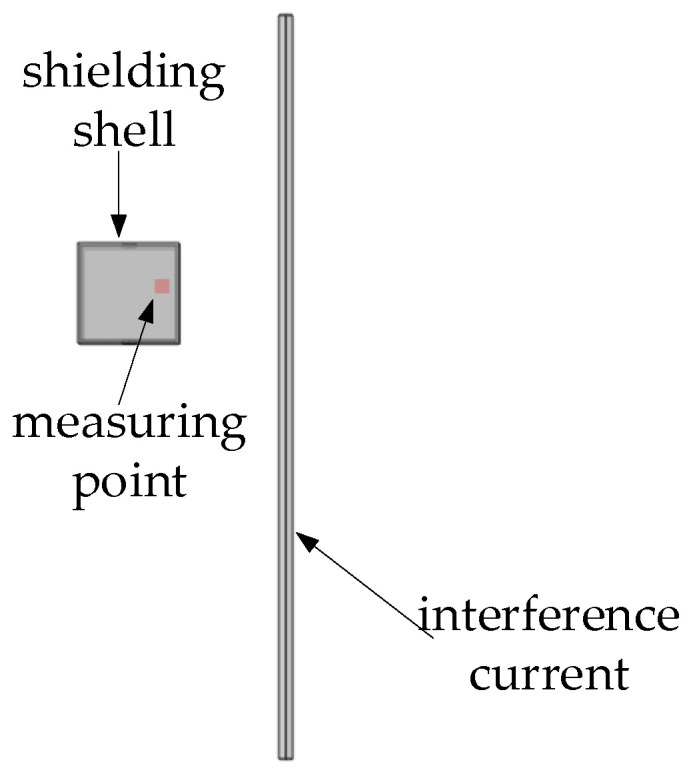
Anti-magnetic interference schematic.

The simulation and analysis of the tunnel magnetoresistive sensor with overlapping shielding layers focus on its anti-interference performance. To capture the electromagnetic interference generated in complex operational conditions within a substation and simplify the interference source model, the simulation defines the interference sources as the spatially uniform magnetic field Bd, simulating geomagnetic field interference, and the current wire IP, simulating interference from other lines. Please refer to Figure 8 for a graphical illustration of this setup.

**Figure 9:**
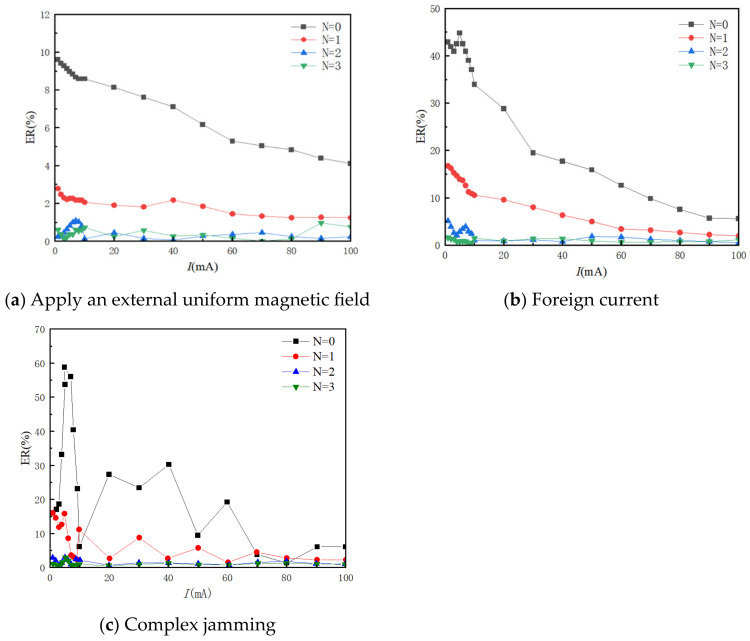
Relative errors of magnetic field measurements under different disturbances.

The simulation and analysis involve applying a 50 Hz AC current, denoted as I, to the current line under test. The current value starts at 1 mA and incrementally increases to 10 mA with a step of 1 mA. It then further increases to 100 mA with a step of 10 mA. Additionally, a power frequency interference current of 10 A is introduced, which is maintained at a distance of 0.05 m from the target line. The investigation focuses on comparing and analyzing the relative error of the magnetic field at the air gap point when faced with different interference sources. The results of the relative error of magnetic field measurement under various interference sources can be seen in Figure 9.

Figure 9 clearly demonstrates that the single magnetic ring structure exhibits weak anti-interference capability when faced with external interference. Both the uniform magnetic field interference and line current interference have a significant impact on the measurement results. This is particularly evident in the case of compound interference, where the maximum relative error of the single magnetic ring structure reaches approximately 62%. Consequently, it becomes challenging to achieve precise measurements in complex substation environments. While the single-layer structure can effectively reduce uniform magnetic field interference, it still falls short in shielding external interference currents when measuring weak currents. Under compound interference conditions, the maximum relative error is 16%. However, as the number of overlapping layers increases, the shielding performance against both types of electromagnetic interference improves gradually. With a double-overlap structure, the maximum relative error is less than 3%, regardless of the magnetic interference. This highlights the superior anti-magnetic capability of the double-overlapping-layer structure. Considering factors such as magnification, anti-magnetic ability, and engineering application, the sensor ultimately adopts the double-overlapping-layer magnetic ring structure. Adopting the double-alternating-layer structure enhances the sensor’s anti-interference ability while maintaining a high magnetic field magnification. This is of utmost significance for accurate measurements in complex electromagnetic environments like substations. The design and manufacturing of the sensor follows the double-alternating-layer structure and undergoes further optimization to meet specific application requirements. This ensures the sensor achieves high accuracy in practical engineering applications.

### 3.3. Design of Residual-Current-Sensing Monitoring System

**Figure 10:**
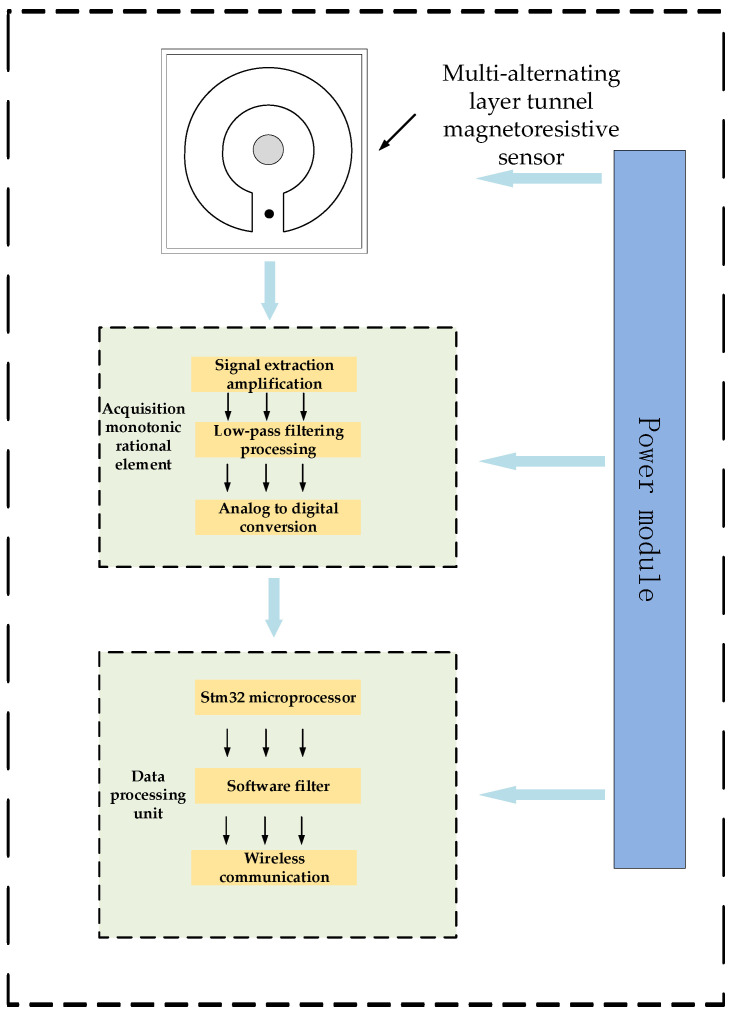
Residual current detection system.

In order to match the magnetic field shielding design scheme designed above, the residual current detection system designed in this section is shown in Figure 10.

The system consists of a Tunnel Magnetoresistive Current Sensor, a high-speed signal conditioning acquisition unit, a data processing control unit, and a power supply and energy acquisition unit. When the sensor is positioned in the measured line, it generates a magnetic field signal, which is then converted into a current signal through an inversion operation of the magnetic field voltage. In the hardware design, the amplitude of the signal output by the sensor needs to be adjusted initially. Following this, the signal undergoes low-pass filter conditioning to ensure that it falls within the acquisition range of the main control chip. Subsequently, the data processing unit utilizes an STM32 to execute the collection command and upload the collected data. The calculated results are then transmitted to the wireless transmission unit via the serial port peripheral device interface, which drives the wireless transmission unit’s communication mode for the final data upload. Finally, to facilitate long-term online monitoring, a power supply is provided to enhance the system’s long-term stable operation.

## 4. Experimental Verification

**Figure 11:**
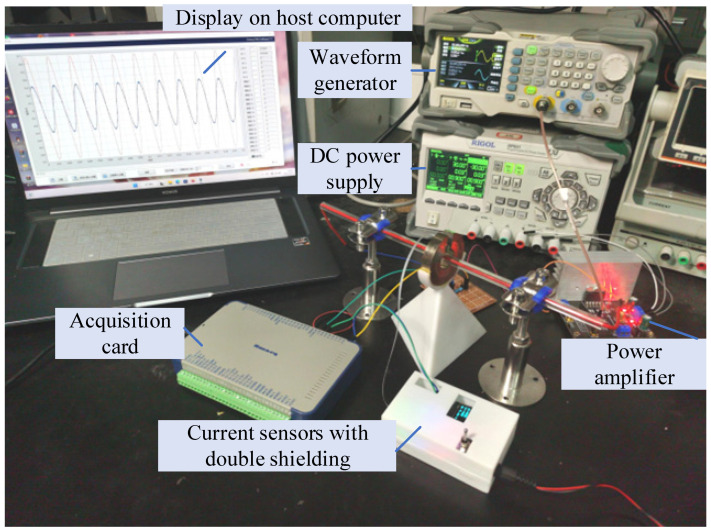
Schematic diagram of test platform.

After the design of the residual current detection system was completed, the test verification was carried out. The purpose was mainly to test the attenuation effect of the magnetic shielding cover on the magnetic field and the residual current detection ability in the face of the AC and DC situations of the substation. The experimental equipment used in the experiment is a computer; a RIGOL DG1032 function generator generates AC and DC bias output voltage signals. The TMR current sensor developed by the TMR2905 module is used to generate the current to be measured and the interference current; a three-dimensional Helmholtz coil is used to simulate the interference of the geomagnetic field of the direct current power supply. The test platform is shown in Figure 11.

### 4.1. Magnetic Shielding Effect Test

The magnetic field interference is observed through the absolute error value, and the absolute error, mean absolute percent error, and root mean square error are used as the evaluation indexes of the magnetic interference performance. Due to the limited measurement time and resources in the laboratory, the evaluation of the stability of the measurement focuses on the assessment of the class A uncertainty of the AC and DC measurements, and the standard deviation MESD is used as the index of the class A uncertainty assessment:

(11) $$e=\frac{I_{test(k)}-I_{true(k)}}{I_{true(k)}}$$

(12) $$\mathrm{MAPE}=\frac{1}{n}\sum_{k=1}^{n}\frac{\left|I_{true(k)}-I_{test(k)}\right|}{I_{true(k)}}\times 100\%$$

(13) $$\mathrm{RMSE}=\sqrt{\frac{1}{n-1}\sum_{k=1}^{n}{\left(I_{true(k)}-I_{test(k)}\right)}^{2}}$$

(14) $$\mathrm{MESD}=\sqrt{\frac{\sum_{i=1}^{n}(\left|e\right|-\bar{\left|e\right|}{)}^{2}}{n-1}}\times 100\%$$

where *I_true_*_(*k*)_ is the actual value of the target current. This value is the exact value at the time of calibration, *I_test_*_(*k*)_ is the measured value of the target current, and *n* is the number of test samples.

**Figure 12:**
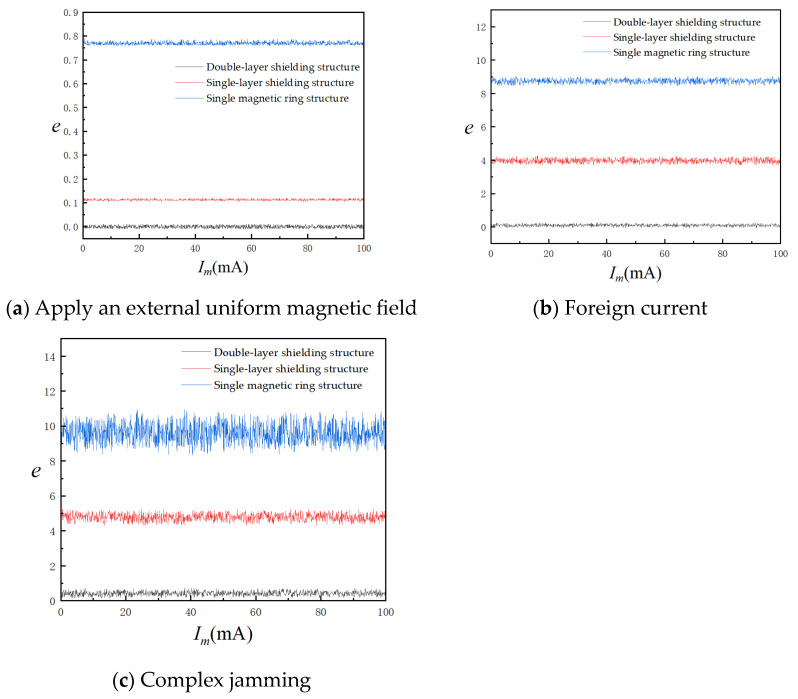
Absolute error for three operating conditions.

In an environment of room temperature 25 °C, the three-dimensional Helmholtz coil was used as a uniform magnetic field generator to simulate the geomagnetic field interference, and the constant-current source output 10 A power frequency AC to simulate the line current interference. The single geomagnetic interference, single line current interference, and composite interference were tested, respectively. The current value to be measured started from 1 mA and gradually increased to 100 mA with a step size of 0.1 mA. A total of 1000 sets of data were recorded. The absolute error *e* curve of the current *I_m_*/mA measured by the tunnel magnetoresistive current sensor under three working conditions is shown in Figure 12.

As depicted in Figure 11, the double-alternating-layer magnetic ring structure demonstrates exceptional anti-magnetic interference capability across various operating conditions in a normal-temperature environment. In the presence of compound interference, the measurement’s absolute error remains within a range of only 0.5 mA. Conversely, the tunnel magnetoresistance current sensor utilizing a single magnetic ring structure is highly susceptible to external magnetic interference, resulting in the highest absolute error values for current measurements. In composite working conditions, the maximum measurement error reaches a significant 10.62 mA, thereby reducing the accuracy of the current measurement. Although the single-alternating-layer magnetic ring structure provides some resistance against the interference of the geomagnetic field, it still exhibits a maximum absolute error of 5.23 mA when confronted with line current interference and compound interference. Consequently, it cannot achieve precise measurements within the complex operating conditions typically found in substations. Table 4 presents the average absolute percentage error and root mean square error data for the three structures of the tunnel magnetoresistive current sensor under compound interference.

The experimental results show that the average absolute percentage error (MAPE) is reduced to 0.239% with the double-alternating-layer polymagnetic ring structure, which is much smaller than 2.534% for the single polymagnetic ring structure and 5.971% for the single-alternating-layer polymagnetic ring structure. The root mean square error (RMS) of the single-polymerized ring structure and the single-alternating-layer polymerized ring structure is 9.671 mA and 4.835 mA, respectively, under the composite interference, which is seriously affected by the magnetic interference. The RMS error of the double-alternating-layer PMR structure is only 0.572 mA. From the point of view of the class A uncertainty characterized by MESD, the double-alternating-layer PMR structure has good stability and certainty. Therefore, in the face of the compound electromagnetic interference conditions in the substation, the proposed double-alternating-layer poly-magnetic ring structure can enhance the anti-magnetic interference ability of the tunnel magnetoresistive current sensor and improve the measurement accuracy of the weak residual current.

### 4.2. AC/DC Performance Test

In this section, we report the performance test of the tunnel magnetoresistive sensor detection system in the face of complex AC and DC residual current situations in substations. In AC or hybrid AC/DC testing, the RIGOL DG1032 function generator was used to generate AC or AC plus DC bias output voltage signals, which were connected to the measuring wire through a power amplifier and a current-limiting resistor to generate AC or hybrid AC/DC waveforms in the measuring wire; in the DC test, the DC power supply was used to stabilize the output DC voltage, and the DC current path was formed by connecting the current-limiting resistance with the measurement wire.

At room temperature of 25 °C, for the current to be measured in the DC and AC tests, we changed the test current to 100 A–1 mA in steps of 100 A, and each group of test currents was repeated five measurements, and the average value was taken to calculate the absolute value of the absolute measurement error, the root mean square error (RMSE), and the uncertainty of class A (MESD). The tunnel magnetoresistive sensing and monitoring system with a double-layer shielding structure and poly-magnetic ring was tested, and the results are shown in Table 5, Table 6 and Table 7 below:

As shown in the tables, the absolute error of the AC measurement is about 1%, the DC measurement error is less than 8%, and the standard deviation of the measurement error is about 1% for current RMS values greater than 300 A in both DC and AC measurements. The class A uncertainty MESD fluctuates between a small range of [0, 1.34]% for both AC and DC measurements, with good stability in general.

**Figure 13:**
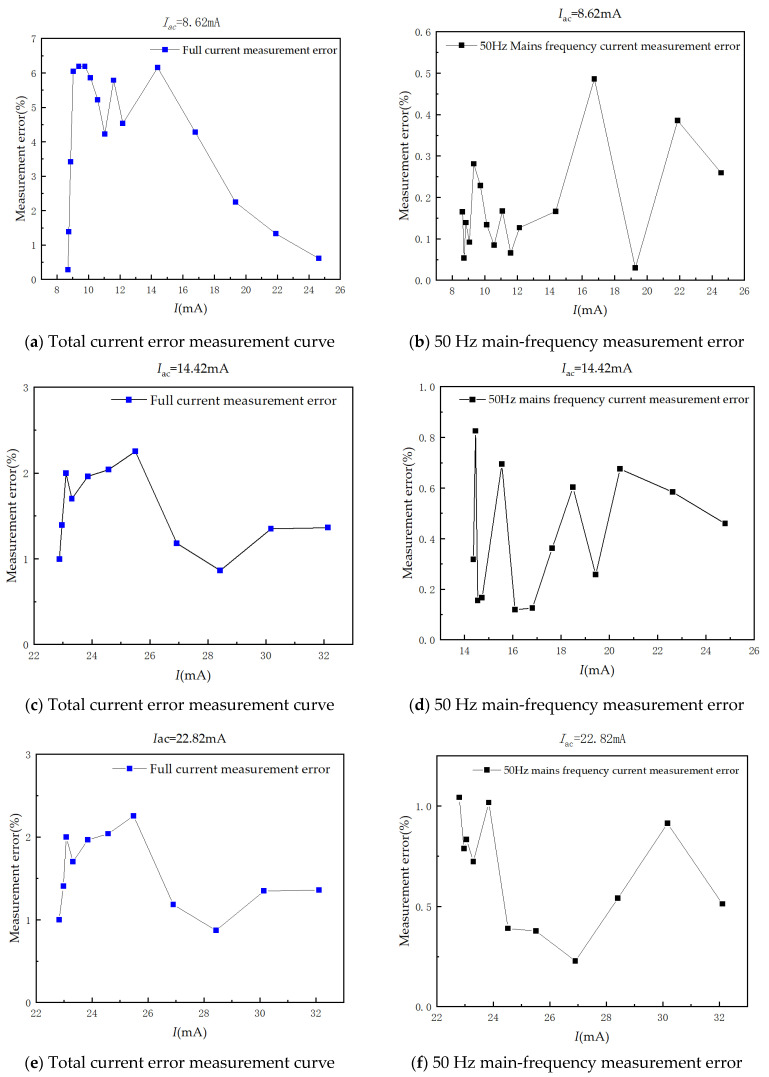
Hybrid AC/DC error curve.

In the AC/DC hybrid test, the effective values of the 50 Hz AC components of 8.62 mA, 14.42 mA, and 22.82 mA are 8.62 mA, 14.42 mA, and 22.82 mA, respectively. The proportion of DC content was changed, and the measurements were performed five times for each group of current, taking the average value and outputting the relative error curve of the full-current amplitude measurement, and finally decomposing the measurement error of the 50 Hz main-frequency current. The results are shown in Figure 13.

The measurement accuracy of the AC component in the AC/DC mixed signal is high, and the measurement error is less than 1%. The full-current measurement error mainly comes from the identification of the DC components. When the AC components are 8.63 mA and 14.42 mA, the waveforms containing different DC components are measured, and the overall measurement error of the sensor is about 6%. When the AC component is 22.82 mA, the measurement error of the designed current sensor for mixed AC and DC is reduced to 2%.

In summary, the measurement system designed in this paper has good response results in DC, AC, or AC/DC measurements, which can meet the residual current detection requirements of substations in the face of complex AC/DC conditions.

## 5. Conclusions

This paper proposes a residual current sensing detection system based on magnetic field shielding. Firstly, by the diversification of the current residual current types and the residual current detection principle used in this paper, it is found that when the tunnel magnetoresistive sensor is used to measure the residual current, it is easily affected by the stray magnetic field of the substation. Therefore, a multi-alternating-layer and magnetic ring combined structure is designed to resist the interference of the external magnetic field. The optimal number of layers of the multi-alternating layers is analyzed. Then, based on this magnetic field shielding structure, the detection system is designed. The final test verifies the magnetic field shielding effect of this system and the feasibility of residual current detection in the case of AC and DC. The main conclusions of this paper are as follows:

1. By using a magnetic ring and a high-permeability alloy shield, the magnetic field is effectively guided so that it can pass through the TMR sensor in a more focused way, thereby improving the accuracy of the measurement. After comparing the magnetic field shielding ability of the single magnetic ring structure, the single-layer alternating structure and the double-layer alternating structure, the double-alternating-layer structure is selected, which can improve the anti-interference ability while maintaining a large magnetic field magnification, which is very important for accurate measurements in complex electromagnetic environments such as substations.
2. The detection system designed in this paper, when detecting AC and DC currents, has a DC measurement error that is less than 1%; the AC measurement error is less than 5%, and the mixed AC and DC measurement error is less than 8%. The designed residual current sensing system has a small error, good response ability, and field measurement stability.
3. This design can improve the accuracy and stability of system measurements, and it can reduce energy waste and improve the overall energy efficiency of the power system by implementing effective measures. Through the accurate measurement of residual current, the abnormal situation of electrical equipment can be determined in advance, which is helpful to prevent accidents. Therefore, it has great potential in the power grid and power system, which can help improve the operation efficiency and safety of the equipment and bring positive benefits to the whole system.
4. For the complex AC and DC waveform measurement, the measurement accuracy is greatly affected by the DC measurement error, and the DC measurement accuracy is related to the noise interference and hysteresis introduction, etc. Subsequently, it is necessary to further study the noise level introduced in each link, design the relevant demagnetization scheme to reduce the interference of the noise on the output signal, and improve the measurement accuracy of the proposed poly-magnetic ring current sensor with multiple alternating layers.

## Figures and Tables

**Table 1:** Characteristics of residual current waveform.

Type	Residual Current Type	Wave Form Characteristic
AC type	AC residual current	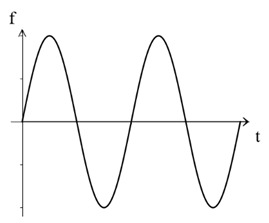
A type	AC residual current	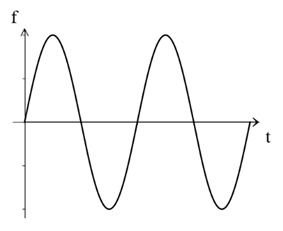
	Pulsating DC residual current	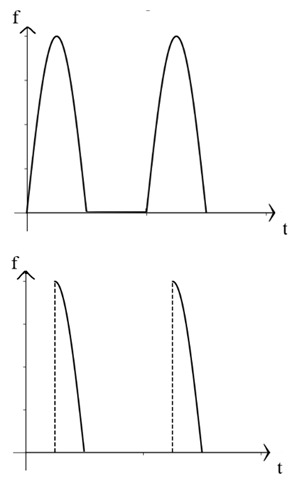
	Half-wave current superimposed on 6 mA smooth DC	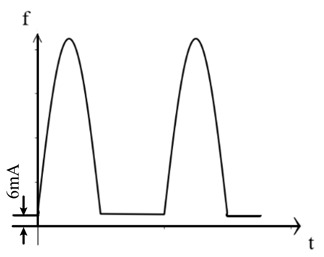
B type	AC residual current	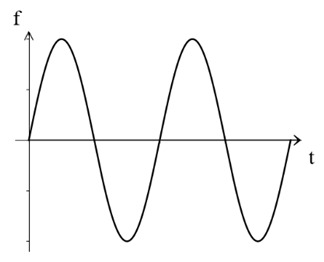
	Pulsating DC residual current	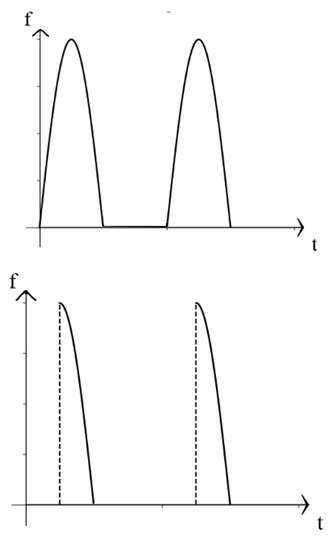
	Half-wave current superposition	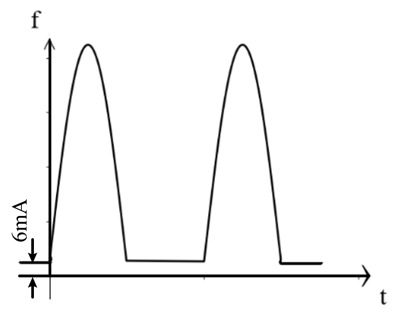
	Smoothing DC residual current	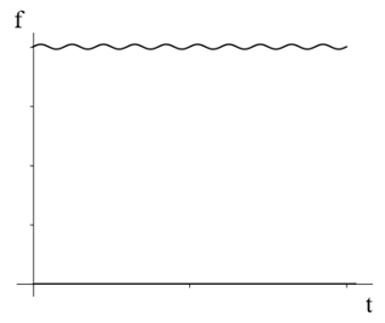

**Table 2:** Material setting.

Name of the Material	Relative Permeability
Aluminum	1
Permalloy	50,000
Copper	1

**Table 3:** Model initialization value.

Model Size Name	Value/mm
r	4
r_1_	10
h	10
d	6
th	1

**Table 4:** Error characteristics of each structure of composite interference.

	Double-Layer Shielding Structure	Single-Layer Shielding Structure	Single Magnetic Ring Structure
MAPE	0.239%	2.534%	5.971%
RMSE	0.572 mA	4.835 mA	9.671 mA
MESD	0.218%	2.478%	5.893%

**Table 5:** Absolute measurement error characteristics in AC/DC case.

Current Amplitude/mA	AC Measurement Error/%	DC Measurement Error/%
0.1	3.21	4.41
0.2	4.87	5.96
0.3	2.64	4.69
0.4	3.18	7.20
0.5	2.95	4.25
0.6	4.2	5.02
0.7	1.09	1.23
0.8	2.05	3.28
0.9	1.31	2.36
1.0	0.52	3.02

**Table 6:** The standard deviation characteristics of relative error in AC and DC cases.

Current Amplitude/mA	Communication Standard Deviation/%	DC Standard Deviation Difference/%
0.1	1.06	1.23
0.2	0.954	1.18
0.3	1.13	0.865
0.4	0.847	0.917
0.5	0.775	0.721
0.6	0.705	0.688
0.7	0.658	0.384
0.8	0.511	0.505
0.9	0.347	0.358
1.0	0.259	0.489

**Table 7:** MESD characterization of class A uncertainty in the AC and DC cases.

Current Amplitude/mA	AC MESD/%	DC MESD/%
0.1	0.987	1.34
0.2	0.932	1.27
0.3	1.07	0.876
0.4	0.861	1.04
0.5	0.732	0.817
0.6	0.714	0.763
0.7	0.675	0.457
0.8	0.549	0.482
0.9	0.382	0.471
1.0	0.281	0.428

## Data Availability

The data that supports the findings of this study are available from the corresponding author upon reasonable request.

## References

[1] Villalobos R.J., Moran L.A., Huenupan F., Vallejos F., Moncada R., Pesce G.C. (2022). A New Current Transducer for On-Line Monitoring of Leakage Current on HV Insulator Strings. IEEE Access.

[2] Ketjoy N., Mensin P., Chamsa-ard W. (2022). Impacts on Insulation Resistance of Thin Film Modules: A Case Study of a Flooding of a Photovoltaic Power Plant in Thailand. PLoS ONE.

[3] Salem A.A., Abd-Rahman R., Al-Gailani S.A., Kamarudin M.S., Ahmad H., Salam Z. (2020). The Leakage Current Components as a Diagnostic Tool to Estimate Contamination Level on High Voltage Insulators. IEEE Access.

[4] Lei M., Peng T., Zhou F., Yu J., Liang S., Liu J., Li L. (2022). Optimal Design and Implementation of Tunnelling Magnetoresistance Based Small Current Sensor with Temperature Compensation. Energy Rep..

[5] Zhang W., Armstrong M., Elgendy M.A. (2019). Mitigation of DC Current Injection in Transformer-Less Grid-Connected Inverters Using a Voltage Filtering DC Extraction Approach. IEEE Trans. Energy Convers..

[6] Vukosavic S.N., Peric L.S. (2017). High-Precision Active Suppression of DC Bias in AC Grids by Grid-Connected Power Converters. IEEE Trans. Ind. Electron..

[7] Vukosavic S.N., Peric L.S. (2015). High-Precision Sensing of DC Bias in AC Grids. IEEE Trans. Power Deliv..

[8] Cubells-Beltran M.D., Reig C., Munoz D.R., De Freitas S.I.P.C., De Freitas P.J.P. (2009). Full Wheatstone Bridge Spin-Valve Based Sensors for IC Currents Monitoring. IEEE Sens. J..

[9] Le Phan K., Boeve H., Vanhelmont F., Ikkink T., De Jong F., De Wilde H. (2006). Tunnel Magnetoresistive Current Sensors for IC Testing. Sens. Actuators A Phys..

[10] Xu X.P., Wang S., Liu T.Z., Zhu M., Wang J.G. (2021). TMR Busbar Current Sensor with Good Frequency Characteristics. IEEE Trans. Instrum. Meas..

[11] Yu J., Yue C., Jiang C., Zhang D., Huang X., Yang C., Li L. (2021). Research on Suppression of External Magnetic Field Interference of Tunnel Magnetoresistive Sensor Based on Versoria Variable Step Improved Adaptive Filtering Method. Energy Rep..

[12] Yang X., Xie C., Wang Y., Wang Y., Yang W., Dong G. (2014). Optimization Design of a Giant Magneto Resistive Effect Based Current Sensor with a Magnetic Shielding. IEEE Trans. Appl. Supercond..

[13] Zhang H., Li F., Guo H., Yang Z., Yu N. (2019). Current Measurement with 3-D Coreless TMR Sensor Array for Inclined Conductor. IEEE Sens. J..

[14] Suwarno, Fari P. Electrical Equivalent Circuits of Outdoor Insulators Based on Leakage Current Waveforms and Computer Simulation. Proceedings of the 2009 IEEE 9th International Conference on the Properties and Applications of Dielectric Materials.

[15] Suda T. (2005). Frequency Characteristics of Leakage Current Waveforms of a String of Suspension Insulators. IEEE Trans. Power Deliv..

[16] Suda T. (2001). Frequency Characteristics of Leakage Current Waveforms of an Artificially Polluted Suspension Insulator. IEEE Trans. Dielectr. Electr. Insul..

[17] Xu X., Tan X., Li W., Ao G., Li X., Xu X., Zhang W. (2022). Research on AC and DC Leakage Detection Technology Based on Fluxgate Principle. Ferroelectrics.

[18] Liu L., Zhang X., Xu L., Luo S., Zhao S., Zhu C., Li S., Hu G., Liu D., Wu J. Direct Current Electronic Current Transformer Based on Current Divider and TMR Sensorr for Use in Direct Current Engineering, Has Combining Unit Providing Laser with Certain Energy Output to Remote Module through Optical Fiber Insulator. https://webofscience.clarivate.cn/wos/alldb/full-record/DIIDW:2022F5438V.

[19] Gobi K., Kannapiran B., Devaraj D. Design of Non-Contact Transduction Based Pressure Sensor Using Tunneling Magnetoresistive (TMR) Principle. Proceedings of the IEEE International Conference on Intelligent Techniques in Control, Optimization and Signal Processing (INCOS).

[20] Zhou J., Zhao W., Peng S., Qiao J., Klein J.-O., Lin X., Zhang Y., Bournel A. (2017). High Tunnel Magnetoresistance in Mo/CoFe/MgO Magnetic Tunnel Junction: A First-Principles Study. IEEE Trans. Magn..

[21] Sun X., Lai P.T., Pong P.W.T. (2014). A Novel Bar-Shaped Magnetic Shielding for Magnetoresistive Sensors in Current Measurement on Printed Circuit Boards. IEEE Trans. Magn..

[22] Malmivuo J., Lekkala J., Kontro P., Suomaa L., Vihinen H. (1987). Improvement of the Properties of an Eddy Current Magnetic Shield with Active Compensation. J. Phys. E Sci. Instrum..

[23] Shao H., Qu K., Lin F., Liang B., Jia K., Ren Q., Li Y., Li W. (2013). Magnetic Shielding Effectiveness of Current Comparator. IEEE Trans. Instrum. Meas..

[24] Cheng Y., Sha Y., Wei S., Bi J., Chang W., Ma X. (2022). Novel Spiral-Gap Shielding Shell for a Rogowski Current Sensor. IEEE Trans. Electromagn. Compat..

